# Evaluation of Lower Limb Muscle Electromyographic Activity during 400 m Indoor Sprinting among Elite Female Athletes: A Cross-Sectional Study

**DOI:** 10.3390/ijerph182413177

**Published:** 2021-12-14

**Authors:** Przemysław Pietraszewski, Artur Gołaś, Michał Krzysztofik, Marta Śrutwa, Adam Zając

**Affiliations:** Institute of Sport Sciences, The Jerzy Kukuczka Academy of Physical Education in Katowice, 40-065 Katowice, Poland; p.pietraszewski@awf.katowice.pl (P.P.); a.golas@awf.katowice.pl (A.G.); martaa.srutwa@gmail.com (M.Ś.); a.zajac@awf.katowice.pl (A.Z.)

**Keywords:** electromyography, muscle activity pattern, lower limbs

## Abstract

The purpose of this cross-sectional study was to analyze changes in normalized surface electromyography (sEMG) signals for the *gastrocnemius medialis*, *biceps femoris*, *gluteus maximus*, *tibialis anterior*, and *vastus lateralis* muscles occurring during a 400 m indoor sprint between subsequent curved sections of the track. Ten well-trained female sprinters (age: 21 ± 4 years; body mass: 47 ± 5 kg; body height: 161 ± 7 cm; 400 m personal best: 52.4 ± 1.1 s) performed an all-out 400 m indoor sprint. Normalized sEMG signals were recorded bilaterally from the selected lower limb muscles. The two-way ANOVA (curve × side) revealed no statistically significant interaction. However, the main effect analysis showed that normalized sEMG signals significantly increased in subsequent curves run for all the studied muscles: *gastrocnemius medialis* (*p* = 0.003), *biceps femoris* (*p* < 0.0001), *gluteus maximus* (*p* = 0.044), *tibialis anterior* (*p* = 0.001), and *vastus lateralis* (*p* = 0.023), but differences between limbs were significant only for the *gastrocnemius medialis* (*p* = 0.012). The results suggest that the normalized sEMG signals for the lower limb muscles increased in successive curves during the 400 m indoor sprint. Moreover, the *gastrocnemius medialis* of the inner leg is highly activated while running curves; therefore, it should be properly prepared for high demands, and attention should be paid to the possibility of the occurrence of a negative adaptation, such as asymmetries.

## 1. Introduction

The 400 m race is considered to be one of the most demanding track and field running events [1]. It is referred to as a prolonged sprint and a “killing event” due to the significant fatigue resulting from the glycolytic effort [2]. Despite the increasing fatigue, the athlete’s goal is to maintain high velocity and skilfully overcome curved and straight sections of the track. During the indoor 400 m event, this is even more demanding as these sections are shorter, resulting in more frequent changes between straights and curves; thus, the ability to adapt to changing running conditions can be extremely desirable.

The times achieved in the curved track sections are significantly slower than those that are straight, due to slower sprint velocities [3]. This is due to the less efficient production of the horizontal ground reaction force, as the athlete uses some of this force to resist centrifugal force [4,5]. Moreover, some studies clearly indicate differences in ground reaction force production between the lower limbs while running curves [4,6,7,8,9]. The left leg (inside) is responsible for stabilizing and directing movement in the frontal plane by braking and changing direction, while the right (outside) leg generates propulsive force and supports the control of movement in the horizontal plane during sprints on curves [4,10]. However, in elite athletes, the medial-lateral ground reaction force increases, but the maximum posterior force is maintained and decreases mainly in weak curve runners, as suggested by Ohnuma et al. [11]. Hence, it seems that in order to maintain similar kinematics and kinetics of movement on curvilinear trajectories as on straight paths, the involvement of specific muscles has to change.

However, thus far, only a few studies have considered this issue [7,12,13]. The latest work by Pietraszewski et al. [13] showed significantly higher normalized surface electromyography (sEMG) signals for the left *gastrocnemius medialis* than for the right leg muscle during the first curve of the 200 m sprint for elite female sprinters in the innermost lane. However, no statistically significant differences were found between the other studied muscles of the lower limbs (*biceps femoris*, *gluteus maximus*, *tibialis anterior*, *vastus lateralis*). The authors speculated that the *gastrocnemius medialis* is the muscle that manages the movement and the distribution of force during sprinting around curves. Yet, it should be noted that just the first curve of the 200 m dash was evaluated. Considering that the 400 m sprint is an extremely demanding event for the athlete, and the fact that fatigue affects the patterns of muscle activity [14,15], it cannot be ruled out that with progressing fatigue, differences in the involvement of specific muscles while running the subsequent curves may occur. To the best of our knowledge, only one study has investigated changes in normalized sEMG signals of the lower limbs during the 400 m sprint [12]. The authors reported greater normalized sEMG signals for the left leg (inside) on the first curve (by ~8%) than for the right leg, but during the run, these asymmetries decreased and, on the second curve, were significantly smaller (by ~3%). Moreover, normalized sEMG signals increased in the next section of the run for both limbs (from 76 to 99% for the right limb, and 82 to 98% for the left limb). It should be noted that the authors studied the 400 m sprint on an outdoor track, meaning their results may differ when compared to an indoor track, where there are more curved sections. Determining whether the engagement of the muscles of the lower limbs differs between subsequent curves on the run may provide valuable insights and practical implications for coaches and athletes that may potentially help athletes avoid overloading individual muscles and improve their performance.

No studies have investigated changes in lower limb muscle activity patterns while elite female sprinters have been running curved sections of the indoor 400 m race. Therefore, the purpose of this study was to analyze changes in normalized sEMG signals of selected lower limb muscles (*gastrocnemius medialis*, *biceps femoris*, *gluteus maximus*, *tibialis anterior*, *vastus lateralis*) between subsequent curved sections of the track during a 400 m indoor sprint. These muscles were selected because their activation is most often analyzed in sprint studies [16]. Based on previous findings, it was hypothesized that the normalized sEMG signals of the *gastrocnemius medialis* will be higher for the left (inside) than for the right leg (outside) while running curved sections of the 400 m race, but the magnitude of this difference will decrease in successive curves. Additionally, it was expected that the normalized sEMG signals of all studied muscles will increase across the 400 m sprint.

## 2. Materials and Methods

### 2.1. Participants

In this cross-sectional study, we analyzed changes occurring in the normalized sEMG signals of selected lower limb muscles in elite female sprinters while they ran subsequent curved sections of the track during a 400 m indoor sprint. The athletes were in the pre-season phase of the annual training cycle. To be included, participants had to be free of neuromuscular and musculoskeletal disorders and report self-determined satisfactory health. In addition, all participants were required to be members of the Polish national team during the last 2 years and to have competed at the national and international level in the previous 2 seasons. All participants were instructed to maintain their normal dietary and sleep habits throughout the study and not to use any supplements or stimulants for 24 h prior to the session. All participants were informed of the objectives and potential risks and benefits of the study prior to giving written informed consent to participate. The research protocol of this experiment received the approval of the Bioethical Committee of the Academy of Physical Education in Katowice (March 2021) and was performed in accordance with the ethical standards of the Declaration of Helsinki, 2013.

A sample size estimation using G*Power software (Dusseldorf, Germany) showed that to detect an effect size of 0.57 [7], the experimental design would require 6 participants to provide 80% power with a significance level of 0.05 and a correlation among repeated measures of 0.5.

### 2.2. Familiarization and Experimental Session

The evaluations were carried out over two trials, 48 h apart (Monday and Wednesday), on an indoor synthetic four-lane track with IAAF certification (Certified Facility by World Athletics as Class 2 [17]). The first session was to familiarize athletes with the experimental procedures; thus, each participant performed one run on the inside lane with sEMG electrodes attached in order to exclude their influence on the quality of the run. During the experimental session, each participant performed a single all-out sprint from a crouched start in the first lane and was instructed to perform the sprint with their habitual pacing strategy. Both trials were performed at the same time of the day (between 9:00 a.m. and 11:00 a.m.) and were preceded by a standardized, sprint-specific warm-up that was consistent with participants’ normal training habits. All 4 curved sections of the track were evaluated, and the radius for the curve on the inside lane was 17.2 m. The participants used their track spikes during the sprint evaluations.

### 2.3. Electromyographic Measurement Procedure

The sEMG signals were recorded bilaterally for the following lower limb muscles: *gastrocnemius medialis*, *biceps femoris*, *gluteus maximus*, *tibialis anterior*, *vastus lateralis.* An eight-channel Noraxon TeleMyo 2400 Wireless system (Noraxon USA Inc., Scottsdale, AZ, USA; 1500 Hz) was used for the measurements and analysis of the biopotentials from the studied muscles. The whole procedure, including maximum isometric voluntary contraction (MVIC) assessment, electrode placement, and normalization of the sEMG signals to the percent of MVIC, was carefully replicated as described elsewhere [13].

### 2.4. Statistical Analysis

All statistical analyses were performed using SPSS (version 25.0; SPSS, Inc., Chicago, IL, USA). The Shapiro–Wilk test was used to verify the normality of the sample data. Two-way ANOVAs with repeated measures were performed to analyze differences in normalized sEMG signals between curved sections and lower limbs during the 400 m sprint (4 curves × 2 sides (right limb vs. left limb)) for each muscle. Effect sizes for main effects and interactions were determined by partial eta squared (η^2^). Partial eta squared values were classified as small (0.01 to 0.059), moderate (0.06 to 0.137), and large (>0.137). Post hoc comparisons using Bonferroni correction were conducted to locate the differences between mean values when a main effect or interaction was found. For pairwise comparisons, effect sizes were determined by Hedges’ g, which was interpreted as ≤0.20 “small”, 0.21–0.8 “medium”, and >0.80 “large”. Statistical significance was set at *p* < 0.05.

## 3. Results

### 3.1. Participants

Ten well-trained female sprinters met the inclusion criteria and participated in the study (age: 21 ± 4 years; body mass: 47 ± 5 kg; body height: 161 ± 7 cm; 400 m personal best: 52.4 ± 1.1 s).

Table 1 and Table 2 show changes in the normalized sEMG signals for selected lower limb muscles while running on the curved sections during the 400 m sprint.

### 3.2. Normalized Surface Electromyography Signals for the Gastrocnemius Medialis

A two-way ANOVA revealed that there was no statistically significant interaction between running curves and the right or left lower limbs (*p* = 0.925; η^2^ = 0.017). The simple main effect analysis showed that running curves (*p* = 0.003; η^2^ = 0.401) and the lower limbs (*p* = 0.012; η^2^ = 0.52) had a statistically significant effect on normalized sEMG signals. Post hoc tests indicated a significantly higher normalized sEMG signal for the left *gastrocnemius medialis* than for the right leg muscle, while running the first and fourth curves (*p* = 0.001, g = 1.37, and *p* = 0.047, g = 0.81, respectively). In addition, there was a significantly higher normalized sEMG signal for the right *gastrocnemius medialis* during the fourth curve in comparison to the first one (*p* = 0.005, g = 1.3).

### 3.3. Normalized Surface Electromyography Signals for the Biceps Femoris

The two-way ANOVA revealed that there was no statistically significant interaction between running the curves and the right or left lower limbs (*p* = 0.23; η^2^ = 0.145). The simple main effect analysis showed that running curves (*p* < 0.0001; η^2^ = 0.559) had a statistically significant effect on normalized sEMG signals, but the lower limbs did not (*p* = 0.388; η^2^ = 0.084). Post hoc tests revealed a significantly higher normalized sEMG signal for the left *biceps femoris* from the second and fourth curves in comparison to the first one (*p* = 0.033, g = 0.73; *p* = 0.007, g = 0.93; *p* = 0.018, g = 1.08, respectively).

### 3.4. Normalized Surface Electromyography Signals for the Gluteus Maximus

The two-way ANOVA revealed that there was no statistically significant interaction between running curves and the right or left lower limbs (*p* = 0.077; η^2^ = 0.221). The simple main effect analysis showed that running curves (*p* = 0.044; η^2^ = 0.255) had a statistically significant effect on normalized sEMG signals, but the lower limbs did not (*p* = 0.486; η^2^ = 0.055). However, post hoc tests did not show any significant differences.

### 3.5. Normalized Surface Electromyography Signals for the Tibialis Anterior

The two-way ANOVA revealed that there was no statistically significant interaction between running curves and the right or left lower limbs (*p* = 0.357; η^2^ = 0.111). The simple main effect analysis showed that running curves (*p* = 0.001; η^2^ = 0.451) had a statistically significant effect on normalized sEMG signals, but the lower limbs did not (*p* = 0.4; η^2^ = 0.08). However, post hoc tests did not show any significant differences.

### 3.6. Normalized Surface Electromyography Signals for the Vastus Lateralis

The two-way ANOVA revealed that there was no statistically significant interaction between running curves and the right or left lower limbs (*p* = 0.629; η^2^ = 0.61). The simple main effect analysis showed that running curves (*p* = 0.023; η^2^ = 0.294) had a statistically significant effect on normalized sEMG signals, but the lower limbs did not (*p* = 0.94; η^2^ = 0.001). Post hoc tests indicated a significantly higher normalized sEMG signal for the right *vastus lateralis* while running the fourth curve in comparison to the third one (*p* = 0.002, g = 0.48).

## 4. Discussion

This study considered the changes in normalized sEMG signals for selected lower limb muscles that occurred between successive curved sections of the track while athletes ran a 400 m indoor sprint. The results of this investigation reveal that the normalized sEMG signals for the studied muscles increased in successive curves during the 400 m indoor sprint. Moreover, the normalized sEMG signals for the left *gastrocnemius medialis* were significantly higher than those for the right leg muscle during the first and fourth curves.

These results confirm part of our hypothesis that the normalized sEMG signals of the *gastrocnemius medialis* will be higher in the left (inside) than in the right (outside) limb while running the curved sections. However, we did not confirm that the magnitude of this difference will decrease over successive curves. Our results show a higher normalized sEMG signal for the left *gastrocnemius medialis* at each curve, and on the first and fourth curves, this was significantly higher than for the right leg. These results are partially in contrast to those reported by Iwanska et al. [12]. Those authors also indicated that higher normalized sEMG signals were noted in the left leg (by ~8%) compared to the right leg while running the first curve; however, this asymmetry decreased at the second curve (by ~3%) during the 400 m sprint on an outdoor track. Nevertheless, it is pertinent that in the current study, the athletes were examined on a 200 m indoor track; thus, the reason for this contradictory finding may lie in the layout characteristics between indoor and outdoor tracks. However, it should be noted that in this study, the athletes ran the first 200 m in the inner lane of the track; thus, the inconsistent results may be explained by differences in track characteristics. Specifically, they may be due to the larger curve radius on the outdoor track (36.5 m) than on the indoor track (17.2 m), making the curve milder [6,18,19]. The results of Chang and Kram [4] partially confirmed these assumptions. Those authors found that the ground contact time increased to compensate for a decrease in vertical ground reaction force as the radius decreased. The results from Pietraszewski et al. [13] may also indicate the size of the curve radius as the cause, as they showed that the normalized sEMG signal for the left *gastrocnemius medialis* was significantly greater than that for the right leg muscle during curve sprinting on the inside lane (lower radius), which was not noticed during sprinting on the outside lane (greater radius).

For the remaining analyzed muscles (*biceps femoris*, *gluteus maximus*, *tibialis anterior*, *vastus lateralis*), a trend of higher normalized sEMG signals of the left than the right limb was observed, but they were not statistically significant. Pietraszewski et al. [13] obtained similar results analyzing a 200 m sprint, and this study showed a similar pattern for a 400 m sprint. Thus, the curved sprint places greater demands on the left (inside) than the right (outside) limb, mainly affecting the *gastrocnemius medialis* muscle. Therefore, these results could confirm suggestions by Pietraszewski et al. [13] that the *gastrocnemius medialis* muscle of the inner limb manages the distribution of forces generated by the remaining muscles.

Furthermore, the gradually increasing normalized sEMG signals of the examined muscles in subsequent curved sections of the track are consistent with literature reports to date [12,20]. It seems that this trend is mainly related to fatigue occurring during the 400 m sprint [21]. A high fatigue level has been shown to occur while running this distance, as revealed by lactate concentrations greater than 20 mmol·L [22], depleted values of phosphocreatine, and considerable decreases in adenosine triphosphate [23]. As a result, over the duration of the 400 m sprint, the contact time increases, the length and frequency of the stride decrease, and the ground reaction force decreases, which translates into a decrease in velocity [24]. This gradual increase in normalized sEMG signals is consistent with studies that have analyzed the effect of fatigue on muscle sEMG signals [12,25,26]. Nummela et al. [25] reported that non-normalized sEMG signals increased significantly during the 400 m sprint, due to the activation of additional motor units to compensate for the ensuing muscle contractility failure caused by metabolic acidosis. Thus, in agreement with Tucker et al. [27], the progressive reduction in running speed is caused by a combination of changes in muscle and regulation of the central nervous system.

Various limitations existed in the present study. First, only elite female sprinters were investigated and only a single indoor running distance was examined; thus, caution should be used when extrapolating these findings to other population samples or conditions. Furthermore, only the curved sections of the evaluated run were studied. Additionally, the external structure of the movement (i.e., ground reaction forces and motion analysis) was not investigated. Moreover, the sEMG signals measured during high-speed movement might be influenced by, for example, electrode shift, changes in the conductivity of the muscle tissue, and crosstalk from other muscles [28]. Lastly, only the peak values for the normalized sEMG signals were analyzed, and we did not consider the abductor muscles. Future research should investigate the entire 400 m sprint (both curved and straight sections) as well as other distances for both males and females.

## 5. Conclusions

In conclusion, the normalized sEMG signals of the lower limb muscles increased in successive curves on the 400 m indoor sprint track. Moreover, the normalized sEMG signals for the *gastrocnemius medialis* muscle of the inner leg were significantly greater than those for the outer leg, and this pattern was maintained throughout the whole race. Coaches and athletes should bear in mind that the *gastrocnemius medialis* of the inner leg is more highly activated while running curves; thus, this could lead to a motor learning effect, and morphological and physiological adaptations in muscle structure and function [29], leading to a risk of overloading, asymmetry, and consequent injury.

## Figures and Tables

**Table 1:** Comparison of normalized sEMG signals of selected posterior thigh muscles while running successive curves on the 400 m track.

Muscle Group	Side	Curve 1	Curve 2	Curve 3	Curve 4
		Normalized sEMG Signals (%MVIC ± SD)			
*Gastrocnemius* *medialis*	Left	160 ± 25 *	182 ± 40	182 ± 48	188 ± 41 *
	Right	127 ± 21	144 ± 30	144 ± 26	159 ± 26 ^#^
*Biceps* *femoris*	Left	97 ± 19	113 ± 23 ^#^	119 ± 26 ^#^	124 ± 28 ^#^
	Right	102 ± 25	110 ± 25	114 ± 23	115 ± 24
*Gluteus* *maximus*	Left	143 ± 28	140 ± 38	153 ± 24	148 ± 30
	Right	127 ± 25	144 ± 24	143 ± 24	151 ± 22

sEMG—surface electromyography; %MVIC—percent of maximum voluntary isometric contraction; SD—standard deviation; * compared with the right limb; ^#^ compared with the first curve.

**Table 2:** Comparison of normalized sEMG signals for selected posterior thigh muscles while running successive curves on the 400 m track.

Muscle Group	Side	Curve 1	Curve 2	Curve 3	Curve 4
		Normalized sEMG Signals (%MVIC ± SD)			
*Tibialis* *anterior*	Left	54 ± 25	60 ± 31	62 ± 32	68 ± 32
	Right	50 ± 27	51 ± 25	55 ± 24	56 ± 26
*Vastus* *lateralis*	Left	64 ± 20	57 ± 15	57 ± 14	62 ± 22
	Right	57 ± 18	60 ± 22	56 ± 24	68 ± 24 *

sEMG—surface electromyography; %MVIC—percent of maximum voluntary isometric contraction; SD—standard deviation; * compared with the third curve.

## Data Availability

The datasets analyzed during the current study are available from the corresponding author on reasonable request.
